# Seroprevalence of Enterovirus 71 Among Children in Western India

**DOI:** 10.3390/v17030356

**Published:** 2025-02-28

**Authors:** Madhu Chhanda Mohanty, Swapnil Y. Varose, Sneha V. Rane, Shailesh D. Pawar, Babasaheb V. Tandale

**Affiliations:** 1 Mumbai Unit, ICMR-National Institute of Virology, Mumbai 400012, Maharashtra, India; ercintswapnil@gmail.com (S.Y.V.); ercintnutan@gmail.com (S.V.R.); shaileshpawarniv@gmail.com (S.D.P.); 2 Epidemiology Group, ICMR-National Institute of Virology, Microbial Containment Complex (MCC) Campus, Pashan 411021, Maharashtra, India; drtandale@gmail.com

**Keywords:** hand-foot-and-mouth disease, EV71, genotypes, seroprevalence, India, antibody, immunity, susceptibility, 1–5-year-old children

## Abstract

Hand-foot-and-mouth disease (HFMD) caused by Enterovirus 71 (EV71) is highly infectious and can lead to serious neurological complications. This study proposed to evaluate the seroprevalence of EV71 in children of two states of western India by estimating neutralizing antibodies (nAbs) to EV71 genotypes D, G, and C isolated in India, using micro-neutralization assay. Among the serum samples of 612 children tested, 213 (34.80%, 95% CI: 31.00–38.73) and 312 (51.00%, 95% CI: 47.00–55.00) were positive for nAbs to EV71 BrCr and indigenous genotype D, respectively, with a significant rise with age for genotype D. However, compared to other age groups, only 23.2% of children aged 1–5 years showed nAbs to EV71 genotype D with a considerably lower Geometric Mean Titer, indicating the susceptibility of this age group to EV71 infection. Our study confirms the circulation of EV71 in India with relatively high susceptibility of children up to 5 years to EV71 infections.

## 1. Introduction

Enterovirus 71 (EV71) is a highly infectious positive-stranded RNA virus that can cause hand-foot-and-mouth disease (HFMD), herpangina, and neurological diseases. Serious complications such as encephalitis, aseptic meningitis, and brain stem encephalitis (BE) may occur in infants and young children. After the poliovirus, it is the second most critical enterovirus in terms of public health. Its prevalence in Malaysia, Taiwan, Singapore, China, and Korea, and continuous worldwide transmission are of global concern [1]. EV71 isolates belonging to various genotypes from cases of acute flaccid paralysis (AFP), HFMD, and encephalitis have been reported in India by ICMR-National Institute of Virology, Mumbai Unit (ICMR-NIVMU) [2,3]. However, there are no reports of outbreaks of HFMD or neuroinfection by EV71, and no systematic studies on the circulation and epidemiology of EV71 in India.

Genomic characterization of EV-A71 strains worldwide has identified seven genotypes, designated A through G. Genotype A is the prototype strain, while Genotype B includes sub-genotypes B0 to B5, and Genotype C comprises sub-genotypes C1 to C4. Genotype D is predominantly found in India and has not been reported elsewhere, similar to Genotype G, which is also unique to India. Genotypes E and F have been reported from Africa. Genotypes D and G are likely endemic to India as they have been isolated from patients with acute flaccid paralysis (AFP) across various regions, although they have not been linked to specific outbreaks. There has been no research to determine if the Indian strains are naturally attenuated, genetically resistant, or have the potential to cause epidemics [4]. The sequence divergence among these genotypes is around 18.99%, highlighting the genetic diversity and rapid evolution of EV71, which underscores the need for continuous surveillance and improved identification methods [5].

In the absence of a readily available vaccine and effective therapy for Indian genotypes, EV71 is a cause of concern regarding acute paralytic disease and associated mortality in children. There is no report on the exposure or seroprevalence of EV71 in Indian children. Therefore, to confirm the circulation of EV71 in India, it was necessary to determine the seroprevalence of EV71 antibodies in Indian children, given the severity of the outbreaks produced by EV71 in the Southeast Asian region.

This study aimed to understand the potential of EV71-caused disease and the possibility of outbreaks in our community. The data have been generated for evidence of population susceptibility or immunity to the EV71 virus, which is of foremost importance in the post-polio eradication era.

## 2. Materials and Methods

### 2.1. Study Design, Ethical Approval, and Serum Sample Collection

This study was a retrospective cross-sectional study. Serum samples collected from children aged 1–15 years for serosurveillance of dengue and chikungunya from the states of Maharashtra and Goa (2009 and 2019) stored in the repository of ICMR-NIV, Pune, India, were used in the study. The seroprevalence studies of dengue and chikungunya were conducted using sera from apparently healthy human participants enrolled randomly through multistage cluster sampling [6]. Details of study participants, such as age, gender, and province of residence, were documented. The approval of the Institutional Ethical Committee (IEC) of ICMR-National Institute of Virology was obtained for retrospective use of serum samples (Human Ethics Letter no. NIV/IEC/June/2019/D-17).

Based on the previously unpublished data [7], the estimated sample size was 250 individuals, considering the seroprevalence of 19% with a 95% confidence level and 5% precision.

A higher sample size of total 612 serum samples was tested using a micro-neutralization assay to estimate neutralizing antibodies (nAbs) against four EV71 strains (prototype strain BrCr genotype A, indigenous genotype D, genotype C, and indigenous genotype G). The serum samples were analyzed as per three age groups—1–5 years, 6–10 years, and 11–15 years (Table 1, Table 2).

### 2.2. EV71 Antibody Testing

Neutralizing antibodies against the prototype strain and genotypes D, G, and C of EV71 were estimated by micro-neutralization assay using Rhabdomyosarcoma (RD) cells [8]. Serial two-fold dilutions (1:8 to 1:1024) of the serum samples were used. All serum samples were tested in triplicate, and positive control serum was used in each assay for quality control. The antibody titer of the serum samples was obtained by finding the lowest dilution at which cytopathic effect was observed in >50% of wells. The titer of >1:16 was considered seropositive for EV71. The antibody titers were calculated using the Reed and Muench method [9]. The possibility of cross-reactivity to other HFMD viruses was ruled out by testing polyclonal antibodies against CVA6 and CVA16 to neutralize EV71 prototype A and genotype D, G, and C viruses.

### 2.3. Statistical Analysis

Statistical analysis was performed to calculate a 95% confidence interval for seroprevalence. Chi-square and Fisher exact tests were performed for comparisons. All statistical analysis was performed using Open EPI (www.openEpi.com) and Sigma Plot Version 10 (Systat Software Inc., San Jose, CA, USA).

## 3. Results

CVA6 and CVA16 antisera did not show cross-reaction with the EV71 virus, indicating that the micro-neutralization assay was specific for the detection of antibodies against the EV71 virus.

### 3.1. Age-Stratified Seropositivity Against EV71 C, D, and G Genotypes

A total of 612 children aged between 1 and 15 years (55.88% male and 44.12% female) were evaluated for nAbs against EV71 genotypes A, C, D, and G (Table 1). Seroprevalence of 53.50% (95% CI 49.10–57.80) and 68.30% (95% CI 64.10–72.30) was found against the prototype strain and genotype D, respectively, in the age group of 6–10 years. The seroprevalence increased as age increased. The increase in seroprevalence of genotypes D, C, and G was statistically significant between 1–5- and 6–10-year age groups (*p* = 0.00004). There was no significant difference observed between 6–10 and 11–15 age groups for any of the three genotypes (C, D, G).

An increasing trend of seropositivity was observed when compared between 1 and 10 years of age; however, above 10 years, the seropositivity rate declined for the EV71-A genotype while no significant change was observed for other genotypes.

**Figure 1:**
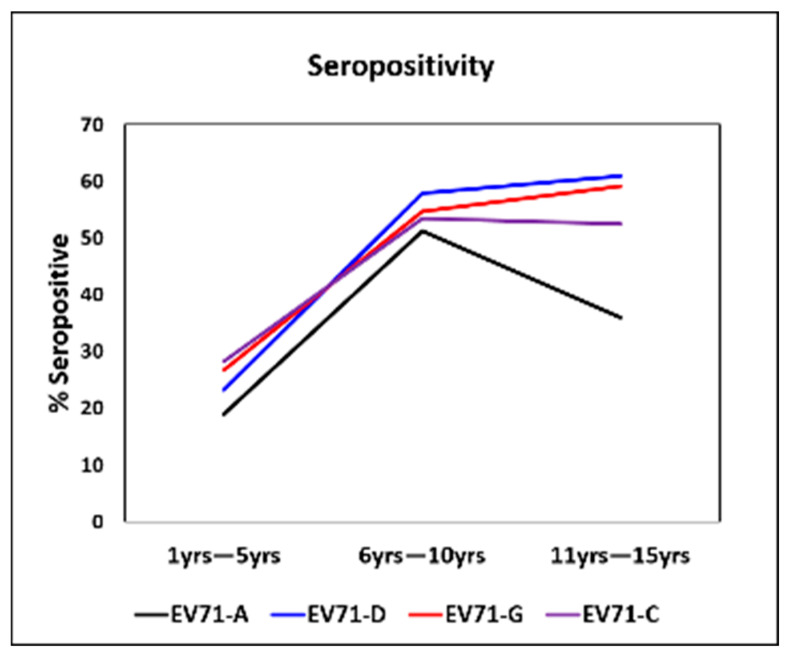
Comparison of seropositivity against EV71 genotypes tested in Indian children. Seropositivity against EV71 prototype A, D, G, and C genotypes tested in Indian children by micro-neutralization assay were compared (*n* = 612). The increase in seroprevalence of genotypes A, D, C, and G was statistically significant between age groups 1–5 and 6–10 years of age by chi-square analysis (*p* = 0.00004).

Seropositivity differed significantly between age groups 1–5 years and 6–10 years (*p* ≤ 0.05), and between 1–5 years and 11–15 years (*p* ≤ 0.05), as compared to between the age groups of 6–10 years and 11–15 years for all three genotypes (Figure 1). Children aged 1 to 5 years had a significantly low seropositivity ranging from 19 to 28 percent.

### 3.2. Percentage Seropositivity to EV71 Genotypes Between Males and Females

**Figure 2:**
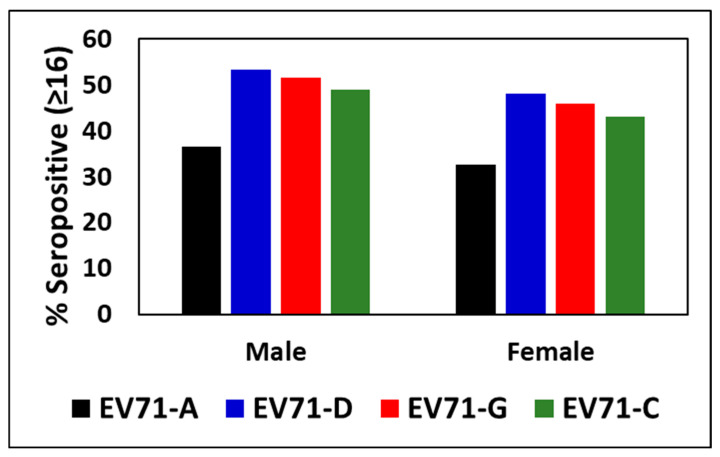
Gender differences in seroprevalence against EV71 genotypes tested in Indian children. No statistical difference could be observed in seroprevalence to EV71 between the males (*n* = 342) and females (*n* = 270) in of the genotypes.

The % seropositivity and GMT of the nAb titer against EV71 genotypes of males (*n* = 372) and females (*n* = 270) were compared to identify any gender specific difference. No significant difference or trend could be found in seropositivity or GMT of antibodies between males and females (Figure 2).

### 3.3. Geometric Mean Titers (GMTs) of Antibodies Against EV71 Genotypes Between the Age Groups

**Figure 3:**
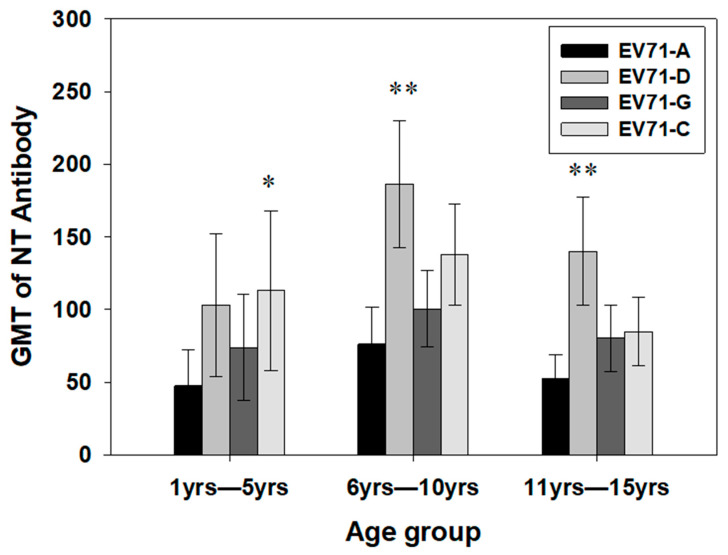
GMT of EV71 genotypes tested in Indian children. GMT of neutralizing antibody titer against EV71 genotypes tested in Indian children (N = 612). The *t*-test for GMT of neutralizing antibody titer against EV71 genotypes was performed at 95% CI. GMT of neutralizing antibodies of age group 1–5 yrs (*n* = 142), 6–10 yrs (*n* = 245), and 11–15 yrs (*n* = 225) were tested. Genotype D showed significantly high GMT in the age group > 6 years (*p* < 0.01, represented by double star symbols, **). Genotype C showed high GMT in the age group less than 5 years (*p* < 0.05, represented by a single star, *). There was a significant difference observed between genotypes A vs. D and C in all three age groups, and in the age group 1–5 years A vs. D (*p* < 0.04) and A vs. C (*p* < 0.05); in the age group 6–10 years A vs. D (*p* < 0.00001), A vs. C (0.00001), and D vs. G (*p* < 0.0001); and in the age group 11–15 years A vs. D (*p* < 0.00001), A vs. C (*p* < 0.02), D vs. G (*p* < 0.00001), and D vs. C (*p* < 0.0001). There was a significant increase in GMT to EV71 D between 1–5-year-old and 6–10-year-old children; also there was a significant decrease in GMT to EV71 C genotype between the 6–10-year-old and 11–15-year-old children.

Genotype D showed a significantly high GMT in the age group >6 years (*p* < 0.01). Genotype C showed a high GMT in the age group <5 years (*p* < 0.05) (Figure 2). The GMT for genotype D increased from 3 years of age, with a peak in the age group 6–7 years and a decreasing trend within 8–15 years of age (Figure 3). In contrast, the GMT of C and G genotypes increased up to 4 years of age and continued to follow a similar trend with a plateau up to 15 years. The percent positivity for the genotypes followed a similar trend for all three genotypes. The GMT of the indigenous EV71-D strain was higher as compared to that of the prototype EV71-A strain. The GMT of genotype D was significantly higher within 6–10 years as compared to other age groups.

### 3.4. Percentage Seropositivity and GMT for EV71 Genotypes at Different Ages

**Figure 4:**
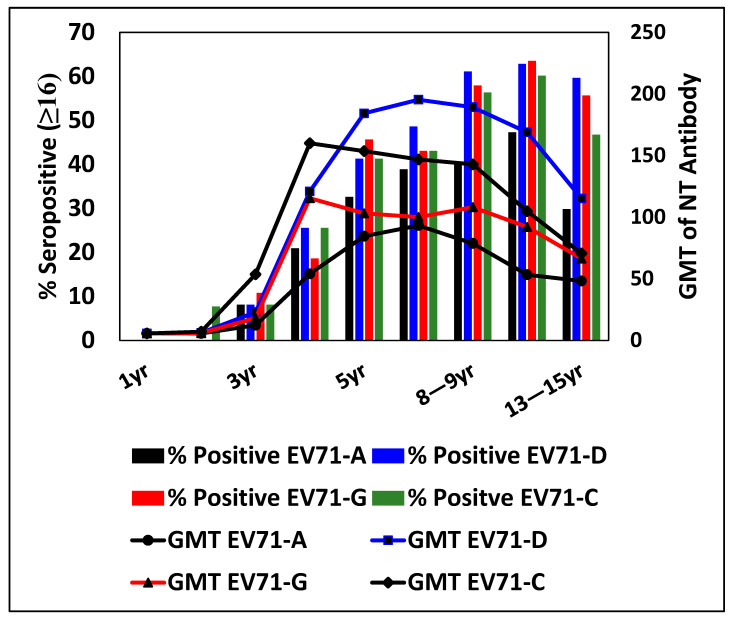
Comparison of percentage seroprevalence and GMT of different EV71 genotypes within the age groups tested in Indian children (N = 612). The % seropositivity for the age group 1–5 years significantly increased in all the EV71 genotypes. Within the genotypes, a statistically significant difference was observed between age groups 2–5 years, 3–5 years, and 4–5 years (*p* ≤ 0.05).

Further analysis of the immune response was performed by classifying nAb titers into four ranges: <1:16 (nil), 1:16–1:32 (low), 1:64–1:256 (moderate), and 1:512–1:2048 (high). For genotype A, 40% of children showed low antibody titers, whereas for genotype D, only 9% were with low titers. The rest of the children showed moderate titers in seropositive subjects. The GMT of all the EV71 genotypes increased up to 4 years of age but decreased after 4 years of age for EV71 G and C genotypes (Figure 3 and Figure 4). Genotype D maintained higher seropositivity and GMT as compared to all other genotypes from 4 to 15 years of age, indicating its prevalence (Figure 4). Interestingly, the GMT of antibodies to genotype D and G was the same at the age group of 4 years, which increased significantly with the increase in age, with a peak at 6–7 years for D, and decreased slowly up to the age of 15 years of age. The GMT against genotypes G and C decreased from 4 years with an increase in age, with a plateau from 4 to 15 years. Children in the lower age group of 1–3 years showed significantly less GMT, which increased at 4 years of age. The GMTs of antibodies against all three genotypes were similar up to 4 years of age, with a comparatively higher GMT of genotype C at the age of 4 years (Figure 3).

Seropositivity between EV71 genotypes A, D, G, and C was compared between age groups from 1 to 5 years of age. The % seropositivity for the age group 1–5 years significantly increased in all the EV71 genotypes. Within the genotypes, a statistically significant difference was observed between age groups of 2, 3, 4, and 5 years (*p* ≤ 0.05). The % seropositive was very low in children up to 3 years of age, irrespective of the genotypes and increased with age for all genotypes. There was no significant difference in % seropositivity within the genotypes.

This indicates that, although the percentage seropositivity to the three Indian genotypes was similar, the GMT varied significantly and was comparatively low compared to the pathogenic strains (C) in the case of the 5–15-year-old age group. In addition, the overall seropositivity and GMT were very low in the age group of 1–5 years, which is vulnerable to HFMD infection.

## 4. Discussion

In the present study, the overall seroprevalence in children up to 15 years was 68.3% (95% CI 64.1–72.3) for genotype D, the major genotype found in India, whereas for the genotypes C1 and G, it was 49.84% and 47.22%, respectively. The overall seropositivity to the three different genotypes did not differ significantly in children up to 15 years of age. Seropositivity increased with age in all three genotypes, whereas it decreased for prototype (BrCr) after 10 years of age. Notably, the 1–5-year-old age group had significantly low seropositivity to all genotypes, which were 23.24%, 26.76%, and 28.17% for D, G, and C genotypes, respectively. Most importantly, the percentage seropositivity was lowest (up to 10%) for children up to 3 years, whereas it was the highest (60%) in the age group of 10–12 years of age, confirming higher exposure in the school-going children.

In the last 15 years, EV71 has caused extensive epidemics of HFMD and occasional neuroinfections in Asia. China, in particular, has reported regular epidemics involving over a million cases since 2008 [10]. EV71 genotypes B4, B5, and C4 are predominantly found in the Asia–Pacific region, while genotypes C1 and C2 are commonly seen in Europe [1]. Genotype C1 of EV71 emerged in Germany in 2015, leading to local outbreaks of severe neurological diseases in France, Poland, and Spain in recent years. During an EV71 outbreak from 2018 to early 2019, Taiwan detected genotype C1 of EV71 in children with HFMD and severe neurological cases [11]. It is reported that the sub-genotype C1 of EV71 circulated only in the Western Pacific, Europe, and the United States before 2000. After 2000, the C1 genotype was prevalent in Southeast Asia, such as Malaysia, Thailand, Hong Kong, and China [12].

In 2001, India reported the first isolation of EV71 from an AFP case [1]. Genotypes D and G have been isolated from AFP cases in India from the wide geographic area. However, outbreaks of EV71 were not reported. EV71 strains of sub-genogroup C1 were isolated in 2011–2012 in India [2]. Sub-genogroup C1 has been linked to cases of AFP, HFMD, and encephalitis. The clustering of C1 isolates with those from Germany, the Netherlands, and Azerbaijan indicates an epidemiologic connection with the EV71 circulating in the European region [2].

The presence of multiple genotypes in India and significant sequence divergence within genotypes D and G indicate that EV71 has been spreading throughout India. Interestingly, human neuronal cells infected with indigenous genotype G induced significantly higher pro-inflammatory cytokines when compared with pathogenic C1 genotypes, indicating the possibility of the G genotype causing neuronal complications. In contrast, the indigenous genotype D did not induce any of the pro-inflammatory cytokines tested [4]. There are no reports of seroprevalence of EV71 in India against any of the circulating genotypes to date. Specific seroprevalence data for sub-genogroup C1 in other Southeast Asian countries are not explicitly available. A total of 41% of serum samples from children ≤ 5 years of age showed high neutralizing activity against a Dutch C1 strain, confirming widespread circulation of EV71 in the Netherlands [13]. The EV71 strains identified in Russia between 2001 and 2011 primarily belonged to subtypes C1 and C2. A study conducted in Russia showed 5 to 20% seroprevalence in children aged 1–2 years and 19% to 83% among children aged 3–5 years. Interestingly, in Asian regions of Russia, elder children had significantly higher seroprevalence rates (41–83%) compared to other regions [14]. In the UK, the seroprevalence of EV71 increased significantly with age. At 6–11 months, only 32% of individuals had antibodies against EV71. By the time individuals reached 10 years of age, over 75% had developed antibodies against EV71 [15]. EV71 is an emerging human pathogen responsible for large-scale outbreaks of HFMD, accompanied by severe neurological complications, in Asia. While EV71 circulates in Europe, it does not cause large outbreaks there. The distinct epidemiological patterns of EV71 infection in Europe and Asia remain an area of interest [12].

According to research conducted in Thailand, 65.8% of people tested positive for EV71-B5. Children aged >6 months to 2 years had the lowest age-adjusted seroprevalence (42.5%), while those aged >6 years had a significant increase to over 80% [16]. However, the percentages were still less than 50% in children less than 3 years old, although antibodies gradually increased with age in all the subjects. Lower seroprevalence in children below 3 years was thought to be the reason leading to the large-scale outbreak of HFMD in 2012 affecting more than 39,000 patients, with an estimated incidence of 62.7/100,000 person-years. Serum samples from Singapore citizens of Chinese, Malay, and Indian origin between 1 and 17 years old were collected during inpatient visits. EV71 antibodies to sub-genogroup B4 had an overall prevalence of 26.9% (95% CI: 24.5–29.5%). From 14.3% in children aged 1–6 to 27.8% in those aged 7–12 and 38.8% in teenagers aged 13–17, it increased dramatically. Between successive age groups, the seroconversion rate fluctuated by about 12%. In a 1996–1997 study, primary school students aged 7–12 had a greater geometric mean titer (GMT) of EV71 antibodies than the 6–12 age group [17].

Studies conducted in endemic countries (where EV71 is prevalent) have consistently shown that seropositivity against EV71 increases with age. In these countries, the lowest seropositivity rates are observed in infants aged 6–11 months and in children younger than 3 years, in whom the seropositivity rates remain below 50% [18]. Although a similar trend has been observed in our study, we are yet to understand the endemicity of EV71 in India due to lack of sufficient information.

In our study, although the seropositivity percentage was similar for all genotypes, the indigenous D genotype had a significantly higher GMT as compared to C and G in the 1–15 years’ age group, and indigenous genotype G had a comparably lower GMT than D and C genotypes. Although percentage seropositivity increases with age for all genotypes, the age-stratified increase in GMT was seen for only genotype D. The GMT decreased with increase in age after 4 years of age for both C and G genotypes, suggesting infrequent re-exposure in older populations. Both seropositivity and GMT are very low or negligible in vulnerable age groups of less than 4 years. The relationship between seropositivity for EV71 and GMT of antibody titers in endemic countries has been reported. Younger age groups (infants) have lower antibody titers. As individuals age, their antibody titers increase. This could be explained by their increased exposure through direct contact with infected individuals, respiratory droplets, indirect contact with contaminated surfaces, and the fecal–oral route as their age advances. Notably, in a study in Thailand, no significant differences were found in antibody titers between different sub-genotypes of EV71 (such as B5 and C4a) or between genders [19]. These findings emphasize the importance of age-related immunity. While seropositivity and antibody titers increase with age, it is essential to continue monitoring EV71 circulation and immunity patterns, considering the longevity of antibodies, to inform public health strategies.

It is well reported that endemic countries experience age-dependent seropositivity and antibody titers against EV71, reflecting the natural course of exposure and immune response, whereas in our study, the GMT decreased for both the C and G genotypes with the increase in age. Interestingly, although the D genotype has not caused any large outbreak, the GMT for the EV71 D genotype increased with age, corroborating with the data of pathogenic genotypes in other endemic countries.

A few studies were conducted in countries that did not report epidemics of neuroinfection like ours. In Germany, one study reported a seroprevalence of 12% at the age of 1–4 years with serum samples collected in 2006 [20], while another study reported a seroprevalence of 27.3% at 0–3 years and 45.6% at 3–6 years with stored samples collected in the year 1997–1998 [21]. Our study reveals up to 10% seropositivity in infants, whereas it increased to 60% in children up to 15 years old. These seroprevalence rates were not noticeably lower than those of the nations that saw severe EV71 virus outbreaks [22]. In Taiwan, EV71 seroprevalence in children was 22–36% before the 1998 epidemic and 24–42% after the epidemic [23,24]. In Singapore, which is located in the endemic region but had only limited outbreaks, seroprevalence among children aged 3–5 years was 30% [25]. In addition, there is no evidence that overt morbidity in endemic regions correlates with seroprevalence [12].

In our study, the seroprevalence of EV71 does not appear to significantly differ between males and females. A similar observation has been reported recently by Girsang et al. 2025, in a study in Indonesia, which quantified EV71 IgG antibodies by ELISA [26]. The overall seroprevalence of EV71 antibodies appears to be influenced more by age than by gender.

India has reported cases of EV71, but large-scale outbreaks have not been as prominent as in some other regions. Our report is the first to document the seroprevalence of EV71 in India, confirming the circulation of its genotypes. The absence of large-scale Enterovirus 71 (EV71) epidemics in India can be attributed to several factors, such as genetic diversity and circulating genotypes, population dynamics, and herd immunity to enteroviruses. Environmental and seasonal factors could also play a significant role. It could be also possible that due to limited severity of the infection, outbreaks are not documented.

The emergence of EV71 is a major concern, especially in Asia. EV71 outbreaks can lead to severe neurological complications, including meningoencephalitis and lung issues like pulmonary edema. Vaccines for EV71 have been developed, but challenges remain, such as genetic variation, standardization, registration, pricing, and surveillance. Continued surveillance and research are essential to monitor EV71 dynamics and disease burden in India [2]. Notably, in the countries with high HFMD prevalence, particularly in mainland China, inactivated EV71 vaccination plays a crucial role in safeguarding children’s health. These vaccines have been confirmed for safety and efficacy by rigorous clinical studies. However, the vaccine strains may vary from region to region and may not provide sufficient protection against other genotypes.

Our study has the limitation of a small sample size in children aged 1 and 2 years. Seroprevalence studies with a large cohort of infants and toddlers in different geographical settings in India are required to understand seropositivity in different age groups. There are a few limitations for seroprevalence studies, which include antigenic similarities between the viruses, making cross-reactivity in serological tests challenging to distinguish between different enterovirus serotypes.

EV71 cyclically caused nationwide epidemics through international importations. It has been shown that seroepidemiology is a poor predictor of EV71 epidemic risk and should be combined with a strong surveillance system. In India, AFP cases are monitored by a very comprehensive WHO National Public Health Support Programme, during which both polio and non-polio enteroviruses are monitored. However, molecular and serological surveillance of EV71 needs to be considered for monitoring the dynamics and impact of EV71 in India.

In summary, our study verifies the presence of various EV71 genotypes circulating in India with a seroprevalence of more than 50% in the age group of 5–15 years, with a very low antibody prevalence in the vulnerable age group of 1 to 4 years. This study highlights the critical need to focus vaccination efforts on younger children, particularly those aged 1 to 4 years, who are most susceptible to EV71 infections. By targeting this high-risk group, vaccination strategies can effectively reduce the incidence of severe disease and potential outbreaks. However, a suitable EV71 vaccine for circulating Indian genotypes needs to be explored or developed, keeping in view any potential outbreak of EV71 in future. Our data underscore the significance of studies on cross-reactive antibodies between the global and Indian genotypes and the efficacy of the available EV71 vaccines in Indian children. In addition, continuous surveillance of EV71 seroprevalence and research with detailed genetic and antigenic characterization are essential to understand the dynamics of EV71 and its disease burden in India. Furthermore, ongoing surveillance of EV71 is vital for prompt outbreak detection and response, ultimately bolstering public health and outbreak preparedness in India.

## Figures and Tables

**Table 1:** Demographic profile of the children of western India included in the EV71 seroprevalence study.

Age Group (Years)	Age in years as Mean (SD)	Male *n* (%)	Female *n* (%)	Total *n*
1–5	3.8 (1.06)	69 (48.6)	73 (51.40)	142
6–10	8.3 (1.29)	131 (53.47)	114 (46.53)	245
11–15	12.9 (1.45)	142 (63.11)	83 (36.89)	225
1–15		342 (55.88)	270 (44.12)	612

**Table 2:** Details of genotypes of the EV71 Indian isolates used for the EV71 seroprevalence study.

EV71 Genotypes	Origin	Year	Age/Sex	Clinical Sample	Clinical Condition	Diagnosis
A (Prototype BrCr)	USA	1969	Infant	Feces	Encephalitis	
G	Jaunpur, Uttar Pradesh	2011	40 m/F	Feces	AFP	Not known
C	Thane, Maharashtra	2012	42 m/M	Cerebrospinal fluid	Encephalitis	Viral Enc
D	Faridabad, Haryana	2001	60 m/M	Feces	AFP	GBS

## Data Availability

The original contributions presented in this study are included in the article. Further inquiries can be directed to the corresponding author.
